# Ultrasound-Based Assessment of Posterior Vitreous Detachment in Healthy Eyes: Clinical and Biometric Factors Associated with More Advanced PVD

**DOI:** 10.3390/jcm14238587

**Published:** 2025-12-04

**Authors:** Cristina Rodriguez-Vidal, Nerea M. Alday, María José Blanco Teijeiro, Manuel Bande

**Affiliations:** 1 Department of Ophthalmology, Hospital de la Santa Creu i Sant Pau, 08025 Barcelona, Spain; 2 Department of Medicine and Surgery, University of Santiago de Compostela, 15782 Santiago de Compostela, Spain; 3 Department of Ophthalmology, Hospital Universitario de Cruces, 48903 Barakaldo, Spain; 4 Department of Ophthalmology, Complejo Hospitalario Universitario de Santiago de Compostela (CHUS), 15706 Santiago de Compostela, Spain

**Keywords:** posterior vitreous detachment, vitreous, B-scan ultrasound, axial length, hypertension, cross-sectional study

## Abstract

**Background/Objectives**: Posterior vitreous detachment (PVD) is an age-related physiological process, yet the clinical and biometric factors influencing its progression remain incompletely characterized in adults undergoing routine ophthalmic evaluation at a tertiary center. Characterizing expected vitreous patterns is essential for interpreting vitreoretinal interface changes in disease. This study aimed to identify independent clinical and biometric factors associated with more advanced PVD stages in adults without macular pathology. **Methods**: In this cross-sectional observational study, 340 eyes from 198 consecutive adults undergoing routine ophthalmological evaluation at a tertiary hospital (March 2022–April 2023) were analyzed. Eyes with current or past macular disease were excluded. Demographic variables, systemic comorbidities and ocular history were recorded. Axial length was measured using optical biometry IOLMaster 700 (Carl Zeiss Meditec, Jena, Germany). Vitreous status was assessed with 10-MHz B-scan ultrasonography and classified as no PVD, partial PVD or complete PVD. Analyses were performed at the eye level. Given its cross-sectional design, this study evaluates associations and cannot establish causal relationships. Bivariate comparisons examined associations between clinical variables and PVD grade. Multivariable modeling was conducted using a clustered generalized estimating equations (GEE) ordinal logit model as the primary analysis, and a secondary independent-eye ordinal logistic regression model was used to evaluate the proportional-odds assumption and model robustness. **Results**: Mean age was 55.6 ± 18.3 years, and 68.5% of eyes were from female participants. No PVD, partial PVD and complete PVD were present in 30.9%, 43.5% and 25.6% of eyes, respectively. In the primary GEE model, axial length (OR 1.35; *p* < 0.001), systemic hypertension (OR 7.13; *p* < 0.001), and prior cataract surgery (OR 2.13; *p* = 0.020) were independently associated with more advanced PVD stages. Age showed a modest but significant association with increasing PVD severity (OR 1.03; *p* = 0.012). Sex and diabetes mellitus were not associated with PVD grade. The independent-eye ordinal model yielded consistent effect directions. **Conclusions**: In adults without macular disease, more advanced PVD stages are independently associated with axial elongation, systemic hypertension, and previous cataract surgery, while age shows a mild but significant association. These findings provide clinically useful contextual reference information for interpreting vitreoretinal interface changes in health and disease. These associations should not be interpreted as causal due to the cross-sectional nature of the study.

## 1. Introduction

Posterior vitreous detachment (PVD) is a common physiological event, closely related to aging and to degenerative changes in the vitreous gel [1]. Progressive liquefaction of the collagen matrix and vitreous syneresis ultimately lead to separation of the posterior vitreous cortex from the inner retina, although the timing and magnitude of this process show marked interindividual variability.

A detailed characterization of the vitreous in clinic-based samples of adults attending routine ophthalmic evaluation is essential to understand the physiological spectrum of age-related changes. Characterizing expected vitreous patterns in healthy adults is essential for interpreting vitreoretinal interface changes in disease. However, most available evidence comes from post-mortem studies or small series using conventional imaging modalities [2]. Advances in in vivo ocular imaging have enabled more precise assessment of vitreous status and the vitreoretinal interface. Among these modalities, dynamic B-scan ultrasonography has become a robust technique to directly visualize the posterior hyaloid and characterize its kinetic behavior. Spectral-domain macular optical coherence tomography (OCT) is, in turn, useful as a complementary tool to exclude macular pathology and to provide additional structural measurements.

Defining the clinical, anatomical and systemic factors associated with PVD in eyes without macular disease is necessary to generate reference values and establish reliable points of comparison. Establishing normal patterns facilitates interpretation of vitreoretinal findings and helps to distinguish physiological aging from potential pathological modulators [3].

The objective of this study was to investigate the clinical and biometric factors associated with increasing PVD severity in a large clinic-based cohort of consecutive adults attending routine ophthalmic evaluation at a tertiary center, without macular pathology.

## 2. Materials and Methods

### 2.1. Study Design

We conducted a cross-sectional observational study aimed at identifying clinical and biometric factors associated with more advanced stages of PVD in a clinic-based cohort of consecutive adults attending routine ophthalmic evaluation at a tertiary hospital center.

### 2.2. Setting and Ethics

The study was conducted at the Department of Ophthalmology, Hospital Universitario de Cruces (Barakaldo, Bilbao, Spain). All examinations and procedures adhered to the tenets of the Declaration of Helsinki (2013 version). The protocol was approved by the local Drug Research Ethics Committee (CEIm OSI Ezkerraldea–Enkarterri–Cruces; approval code E22/11; approval date: 29 March 2022). Written informed consent was obtained from all participants prior to inclusion.

### 2.3. Participants

A total of 340 eyes from 198 adult subjects (≥18 years) consecutively evaluated between March 2022 and April 2023 were included. Eyes with current or previous macular pathology, including age-related macular degeneration (AMD), history of vitreoretinal surgery, or active inflammatory or neoplastic ocular disease were excluded.

### 2.4. Sample Size Justification

A priori sample size estimation was performed following methodological recommendations for ordinal multivariable models, which suggest a minimum of 10–15 outcome events per variable [4]. With 7 planned covariates, the required number of eyes in the least frequent category (complete PVD) ranged from 70 to 105. Based on prior literature reporting a 20–25% prevalence of complete PVD in healthy adults [2], the total sample needed was estimated at 280–350 eyes. Our final cohort included 340 eyes, with 87 complete PVD cases, thereby meeting the predefined requirements and ensuring adequate model stability and low risk of overfitting. Because ordinal logistic models require defining the limiting category for events-per-variable calculations, the least frequent outcome level (complete PVD) was used as the ‘event’ category for determining the minimum sample size.

### 2.5. Variables and Procedures

At baseline, all subjects underwent detailed medical history and comprehensive ophthalmic examination. Demographic variables (age, sex, laterality), physical status according to the American Society of Anesthesiologists (ASA), relevant systemic comorbidities (arterial hypertension, diabetes, connective-tissue disorders), and detailed ophthalmic history were recorded. Previous ocular surgeries—including exclusively uncomplicated phacoemulsification in pseudophakic eyes—were documented; the timing of cataract surgery relative to ultrasonography was recorded as part of the ophthalmic history but was not used as an inclusion or exclusion criterion. High myopia, prior laser treatments, and previous uveitis were also documented. Because systemic comorbidities were inherently patient-level characteristics, each condition (arterial hypertension, diabetes mellitus, and collagen vascular disease), as well as ASA physical status, was recorded once per patient and subsequently assigned to both eyes in bilateral cases. Hypertension status was extracted from the electronic medical record, based on prior documented diagnosis and current antihypertensive treatment; no self-reported hypertension was used. Blood pressure was not re-measured at the study visit, and therefore contemporaneous measurement was not incorporated.

High myopia was not used as an exclusion criterion. High myopia was defined as a spherical equivalent ≤−6.00 diopters and was recorded for all phakic eyes as part of the ophthalmic history. However, refractive error was not included as a covariate in the multivariable models because it was not a primary focus of the analysis and was partially captured by axial length. Other non-macular ocular pathologies were handled as follows: eyes with previous vitreoretinal surgery, ocular inflammation, or any media opacity impairing image acquisition were excluded a priori, whereas eyes with phakic lens opacity were included. Lens status (phakic vs. pseudophakic) was incorporated into the multivariable analysis through the variable “previous cataract surgery.” Although data on refractive error were available, detailed grading of phakic lens opacity was not systematically collected, preventing its inclusion in sensitivity analyses. Therefore, lens status was operationalized as “phakic vs. pseudophakic,” and prior cataract surgery was included as a covariate in the multivariable models.

Axial length was measured using optical biometry (IOLMaster 700, Carl Zeiss Meditec, Jena, Germany). Best-corrected visual acuity (BCVA) was recorded using a Snellen chart and subsequently converted to LogMAR units for statistical analysis.

Spectral-domain macular optical coherence tomography (Spectralis OCT, Heidelberg Engineering, Heidelberg, Germany) with LINE and CUBE protocols was performed to quantify foveal thickness and confirm absence of macular disease.

PVD status was evaluated using dynamic B-scan ultrasonography with a 10-MHz probe (Quantel Compact Touch, Clermont-Ferrand, France). Static and dynamic (K-scan) exploration was performed to classify the posterior hyaloid in three categories: (1) no PVD, (2) partial PVD, and (3) complete PVD.

### 2.6. Ultrasound-Based Assessment of PVD

PVD was assessed using standardized ocular ultrasonography (Quantel Compact Touch, 10-MHz probe). After instillation of topical anesthesia, the probe was placed directly on the bulbar conjunctiva using sterile ophthalmic gel as the coupling medium.

For each eye, six dynamic B-scan projections were obtained in a systematic fashion: four transverse (superior, inferior, temporal, nasal) and two longitudinal (temporal and nasal). Real-time kinetic assessment in primary position and during small eye movements allowed evaluation of posterior hyaloid mobility and detection of residual vitreoretinal adhesions.

**Figure 1:**
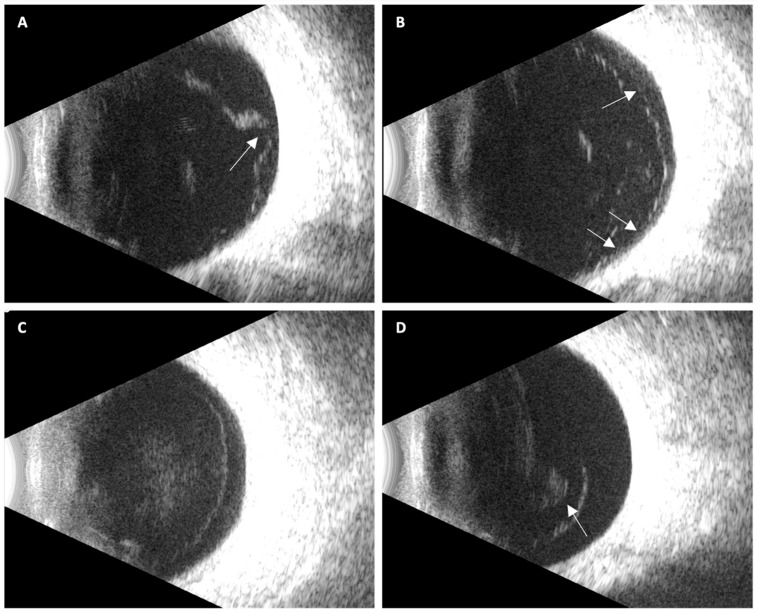
Representative ultrasound images illustrating posterior vitreous detachment (PVD) stages: (**A**) Localized focal partial PVD, with persistent vitreoretinal adhesion at the optic disk (arrow); (**B**) Broad incomplete PVD showing multiple residual adhesion points, resulting in partial posterior hyaloid mobility (arrows); (**C**) Complete PVD without vitreous collapse, characterized by a uniformly detached, immobile, hyper-echoic membrane with a convex configuration; (**D**) Complete PVD with vitreous collapse, with a visible Weiss ring (arrow).

PVD status was categorized using a predefined three-stage ultrasound system (Figure 1):

- No PVD: no identifiable hyaloid separation; homogeneous vitreous cavity.
- Partial PVD: localized or broad separation with persistent adhesion at the macula and/or optic disk.
- Complete PVD: hyper-echoic membrane full detachment of the posterior hyaloid from the optic nerve head and macula, visible in all projections, with or without a Weiss ring.

This operational system is consistent with established models of age-related PVD progression and prior descriptions of its early stages [5,6,7].

### 2.7. Ultrasound Image Evaluation

Ultrasound images were independently assessed by two evaluators (C.R-V and M.B.) using the predefined three-stage classification. In cases of disagreement, a senior adjudicator (M.J.B-T.) reviewed the images and provided the final decision. Interobserver agreement was excellent, with only three discrepant classifications out of 340 evaluations (Cohen’s kappa = 0.986; 95% CI 0.96–0.997).

### 2.8. Unit of Analysis

The analysis was performed at the eye level. In bilateral cases, both eyes were considered independently.

### 2.9. Statistical Analysis

Continuous variables were reported as the mean ± standard deviation. Between-group comparisons were conducted using Student’s *t* test, the Mann–Whitney U test, or Pearson’s χ^2^ test, according to the distribution and nature of the data. The primary multivariable analysis used a clustered generalized estimating equations (GEE) ordinal logit model, with an exchangeable working correlation structure to account for within-subject dependence between fellow eyes. Because fellow eyes within the same participant are not statistically independent, an exchangeable intra-subject correlation structure was assumed, and a population-averaged GEE ordinal logit model was used as the primary analysis to appropriately account for within-subject dependency. Independent-eye models were retained only as secondary sensitivity analyses. Robust (sandwich) standard errors were applied, and effect sizes were expressed as odds ratios with 95 percent confidence intervals. Because GEE does not produce likelihood-based R^2^ statistics, model adequacy was assessed using the quasi-likelihood under the independence model criterion (QIC) and its complexity-corrected version (QICC).

As a secondary sensitivity analysis, we also fitted a conventional independent-eye ordinal logistic regression model (cumulative logit) to explore whether any violations of the proportional odds assumption or clustering could influence the results.

Prior to multivariable modeling, multicollinearity among predictors was evaluated using the Variance Inflation Factor (VIF) and correlation matrices. All predictors showed VIF values < 2.2 (Edad 2.13; HTA 1.96; prior cataract surgery 1.44; AXL 1.14), indicating absence of problematic collinearity. No pairwise correlations exceeded thresholds of concern. Therefore, all variables were retained in the final model.

Statistical significance was defined as *p* < 0.05. No correction for multiple testing was applied because the analyses were based on a single prespecified multivariable model rather than on multiple independent hypothesis tests. All descriptive and univariable analyses were performed using IBM SPSS Statistics^®^ version 28.0 (IBM Corp., Armonk, NY, USA). The clustered GEE ordinal logit model was computed using R software (version 4.2.0; R Foundation for Statistical Computing, Vienna, Austria).

## 3. Results

A total of 340 eyes without macular disease from 198 patients were analyzed. These were consecutive participants evaluated at the Ophthalmology Department of Hospital Universitario de Cruces (Barakaldo, Spain). Among them, 56 were unilateral cases and 142 were bilateral.

### 3.1. Descriptive Analysis

The cohort was predominantly female, with 233 eyes (68.5%) from women and 107 eyes (31.5%) from men. At the patient level, this corresponded to 135 women (68.2%) and 63 men (31.8%). Mean age was 55.5 ± 18.2 years (median 57; range 18–90), illustrating the broad age distribution of the sample.

In the biometric assessment, mean axial length measured 23.66 ± 1.46 mm (range 20.97–31.77 mm). Mean baseline foveal thickness was 277.08 ± 31.93 µm, while mean BCVA on the Snellen scale was 0.85 ± 0.26, indicating generally preserved visual function.

Regarding ophthalmic history, most patients (160/198; 80.8%) had no history of cataract surgery, corresponding to 273 eyes (80.3%). With respect to systemic comorbidities at the patient level, 99 patients (50.0%) had arterial hypertension and 28 patients (14.1%) had diabetes, while collagen vascular disease was present in 9 patients (4.5%). In the eye-level dataset, this translated into 69.7% of eyes (237 eyes) from individuals without hypertension, 11.5% (39 eyes) with diabetes, and 4.7% (16 eyes) with collagen disease. According to the ASA classification, 125 patients (63.1%) were classified as ASA 1, 67 (33.8%) as ASA 2, and 6 (3.0%) as ASA 3, closely mirroring eye-level proportions (61.5%, 36.2% and 2.4%, respectively).

Regarding vitreous status, 105 eyes (30.9%) showed no posterior vitreous detachment, 148 eyes (43.5%) exhibited partial detachment, and 87 eyes (25.6%) demonstrated complete detachment (Table 1).

### 3.2. Demographic and Clinical Comparison

The comparative analysis across vitreous status groups demonstrated consistent and clinically meaningful differences. Age increased markedly with advancing vitreous detachment, from 47.0 ± 15.0 years in eyes without PVD to 53.9 ± 18.6 years in those with partial PVD and 68.5 ± 13.3 years in eyes with complete PVD (*p* < 0.001). Axial length also differed significantly between groups (*p* = 0.022), ranging from 23.37 ± 1.30 mm in the no-PVD group to 23.68 ± 1.24 mm in partial PVD and 24.06 ± 1.90 mm in complete PVD.

Best-corrected visual acuity (Snellen) appeared lower in eyes with more advanced PVD in the unadjusted analysis (*p* < 0.001), decreasing from 0.92 in eyes without PVD to 0.74 in those with complete detachment. However, this gradient is likely driven by age-related factors and lens status rather than by vitreous stage itself, as suggested by the lack of an independent association in the multivariable clustered model. In contrast, foveal thickness did not differ significantly among groups (*p* = 0.966), indicating that vitreous status did not meaningfully affect central retinal thickness in this cohort.

Sex distribution also varied by group, with a higher proportion of females in the no-PVD category (81.0%), reaching statistical significance (*p* = 0.004). Systemic factors showed clear associations with vitreous status: hypertension was substantially more prevalent in eyes with complete PVD (67.8%; *p* < 0.001), and diabetes likewise demonstrated a significant gradient across groups, with the highest prevalence in complete PVD (21.8%; *p* = 0.002) (Table 2).

### 3.3. Multivariate Analysis

Because several participants contributed both eyes, the primary multivariable analysis was performed using a clustered generalized estimating equations (GEE) ordinal logit model, applying an exchangeable working correlation structure to account for within-subject dependence and robust sandwich standard errors.

In the GEE model, age, axial length, systemic hypertension and prior cataract surgery were independently associated with increasing PVD severity. Each additional year of age was associated with approximately 3% higher odds of being in a more advanced PVD category (OR ≈ 1.03; 95% CI 0.95–0.99; *p* = 0.012). Longer axial length likewise increased the odds of more advanced PVD (OR ≈ 1.31; 95% CI 1.11–1.53; *p* = 0.001). Hypertension showed the strongest association (OR ≈ 7.0; 95% CI 2.6–19.0; *p* < 0.001), and prior cataract surgery was also significant (OR ≈ 2.5; 95% CI 1.3–4.7; *p* = 0.004). In contrast, sex, diabetes, ASA status and baseline BCVA were not significantly associated with PVD severity after accounting for clustering. Clinically, these effect sizes indicate that each additional millimeter of axial elongation confers a 30–35% increase in the odds of being in a more advanced PVD stage, previous cataract surgery approximately doubles the odds, and systemic hypertension increases the odds by nearly seven-fold. These values help contextualize the relative magnitude of each factor’s association.

Model adequacy was evaluated using QIC and QICC, both indicating good overall fit. Full GEE results are provided in Table 3.

As a secondary sensitivity analysis, we additionally fitted a conventional independent-eye proportional-odds model. Although this model produced similar effect directions, formal tests showed deviations from the proportional-odds assumption for several predictors, further supporting the use of the GEE framework as the primary analytic approach.

## 4. Discussion

These results allow us to define the behavior of the vitreous gel in the healthy human eye throughout adult life. Characterizing expected vitreous patterns is important for providing a clinical framework to interpret vitreoretinal findings, although establishing true population-wide reference values would require community-based sampling rather than a tertiary-care cohort. In addition, the identification of clinical factors associated with more advanced PVD stages contributes to understanding which elements may modulate the speed and manner in which posterior hyaloid detachment occurs, distinguishing physiological aging from potential pathological influences.

The sample included 340 eyes from 198 patients without known macular pathology. Mean age was 55.5 ± 18.2 years, with a range from 18 to 90 years, which provides a broad cross-sectional perspective of physiological vitreous changes across the lifespan. This age breadth makes it possible to observe the vitreous from youth—compact and firmly adherent—through to the degenerative changes present in advanced stages of life.

Sex distribution showed a predominance of females (68.5%). This pattern has been reported in many clinical ophthalmology settings and may reflect higher attendance of women to routine eye-care services, although this was not specifically assessed in our study. This observation aligns with the clinic-based nature of our sample and should not be interpreted as evidence of sex-related biological differences in vitreous aging. Moreover, the study was not powered to detect sex-specific effects, and therefore no conclusions about sex-related differences in PVD progression can be drawn. Likewise, there was a considerable prevalence of comorbidities such as hypertension (30.3%) and diabetes mellitus (11.5%), systemic diseases widely recognized for affecting ocular microcirculation and altering extracellular matrix metabolism. These factors could accelerate vitreous degeneration through chronic inflammatory mechanisms, endothelial damage, and oxidative stress [8].

One of the most relevant findings was the strong relationship between age and vitreous status. Patients without signs of PVD had a mean age of 47 years; those with partial PVD, 54 years; and those with complete PVD, 68 years. This clear gradient reflects the expected physiological evolution of the vitreous and reinforces age as the primary factor associated with posterior vitreous separation, a relationship that also remained significant in our multivariable and clustered analyses. This pattern has been consistently described in the literature for more than half a century. Favre and Goldmann [9] already noted that PVD was exceptional before age 45, but its frequency increased significantly after the sixth decade of life. Foos [10], in his post-mortem study of more than 700 globes, found a prevalence of 41% PVD in individuals older than 65 years. These findings are supported by those of Kishi et al. [3] and Uchino et al. [2], who documented the progressive processes of liquefaction and syneresis that culminate in separation of the posterior vitreous cortex from the retina. Our data also align with those reported by Hikichi et al. [11], who observed a mean age of 65 years in patients with complete PVD. Ripandelli et al. [12], in a longitudinal study, detected PVD signs in 63% of individuals over 60 years of age. Taken together, these results reinforce the hypothesis that aging is the main driver of structural vitreous collapse. From a molecular perspective, Sebag et al. [13] described that these changes are mediated by the progressive degradation of type II collagen and hyaluronic acid (HA), the main components of the vitreous gel. Loss of these macromolecules leads to reduced cohesion and transparency, promoting central vitreous liquefaction and peripheral contraction of its matrix.

Beyond age, we identified a significant association between PVD and axial length. In our sample, patients with complete PVD had a mean axial length of 24.02 mm, compared with 23.71 mm in partial PVD cases and 23.42 mm in those without PVD. This pattern is consistent with previous research such as that of Yonemoto et al. [14], who proposed that longer eyes are subjected to greater anteroposterior traction, potentially facilitating vitreous detachment through chronic mechanical stress at the vitreoretinal interface. This phenomenon is particularly relevant in the context of axial myopia, where ocular elongation can modify vitreous architecture and its adhesion points to the retina. Future studies should further explore the interaction between axial length, degrees of myopia, and the temporal sequence of PVD. Although our sample included relatively few highly myopic eyes, the observed association suggests that ocular elongation, even within non-pathologic ranges, may influence vitreous detachment dynamics.

BCVA showed a progressive decrease with advancing PVD: from 0.92 in eyes without PVD to 0.85 in eyes with partial PVD, and 0.74 in cases of complete PVD. This finding is consistent with studies suggesting that vitreous liquefaction, the appearance of opacities or syneresis, and the formation of vitreous lacunae can interfere with light transmission, reducing optical quality even in the absence of macular disease [15,16]. However, it is important to consider that the association between PVD and decreased BCVA may be mediated by advanced age. In this sense, associated pathologies such as cataracts may act as confounding factors. The Age-Related Eye Disease Study (AREDS) [17] and the works of Ripandelli et al. [12] and Yonemoto et al. [14] highlight the need to adjust this analysis for lens status. Accordingly, the BCVA gradient observed in unadjusted comparisons likely reflects confounding by age and pseudophakia, which is consistent with the absence of an independent association in the multivariable clustered GEE model.

Regarding foveal thickness, no statistically significant differences were found between the different degrees of PVD. This finding is consistent with what was described by Uchino et al. [2], who reported that in the absence of significant traction, foveal thickness remains relatively stable. Complementarily, studies such as those of Ito et al. [18] and Gupta et al. [19] described foveal thinning in scenarios of marked vitreous traction or advanced macular pathology. Therefore, in populations without retinal disease such as the present one, foveal thickness appears to be a variable with low sensitivity to physiological vitreous changes.

The comorbidity analysis revealed a cross-sectional association between systemic diseases and vitreous status. Arterial hypertension was more prevalent in the complete PVD group; however, due to the cross-sectional design, this finding should be interpreted as an association rather than evidence of causality. Numerous studies have shown that hypertension alters retinal microvasculature, generating endothelial dysfunction, increased permeability, and weakening of the blood-retina barrier [20,21,22,23]. This alteration favors infiltration of inflammatory mediators into the vitreous, which can accelerate its liquefaction and promote PVD [24]. These findings suggest that control of chronic vascular risk factors may have an indirect but relevant impact on vitreous structural health, opening a potential preventive intervention pathway in patients at risk of vitreoretinal diseases.

The multivariable ordinal logistic model identified age, axial length, systemic hypertension and previous cataract surgery as the strongest independent variables linked to increasing PVD severity. Although GEE models do not yield traditional or pseudo–R^2^ measures, their population-averaged estimates are appropriate for paired-eye data, and the QIC and QICC values indicated stable and coherent model performance for the selected factors associated with PVD severity. Within this framework, age remained a significant factor associated with PVD severity, reinforcing its central role in the physiological progression of vitreous separation. Notably, previous cataract surgery showed a robust association with more advanced PVD, consistent with earlier reports suggesting that lens extraction may alter intraocular biomechanical forces and weaken vitreoretinal cohesion. This finding aligns with previous research by Hilford et al. [25], Ivastinovic et al. [26], and Ripandelli et al. [12], all of whom described an increased incidence of PVD following phacoemulsification. Mechanistically, cataract surgery may facilitate posterior hyaloid detachment by modifying vitreous hydration dynamics and reducing cortical adhesion, particularly in eyes already predisposed by age or axial elongation. The attenuation of the age coefficient in the GEE model likely reflects shared variance with hypertension and pseudophakia, both strongly age related. This should not be interpreted as contradicting the well-established role of aging in PVD physiology, but rather as indicating that, once clustering and correlated covariation are considered, systemic vascular status and prior lens extraction emerge as the most prominent independent contributors to PVD advancement in this sample.

Describing vitreous patterns in healthy adults is clinically relevant, while acknowledging that true population-wide values would require broader community-based sampling. In routine practice, vitreoretinal interface findings often require interpretation in contexts where the biological behavior of the posterior hyaloid is uncertain. Characterizing expected trajectories of PVD across adult life allows clinicians to better distinguish physiological aging from potential modifiers. Moreover, the identification of clinical factors associated with more advanced PVD stages contributes to understanding which elements may modulate the speed and manner in which posterior hyaloid separation occurs. Accordingly, these results provide a baseline framework that may help contextualize vitreous status in future studies of disease, intervention or longitudinal change.

### Study Limitations and Future Research

Several limitations should be acknowledged. First, although the multivariable model identified strong and consistent factors associated with vitreous status, a substantial proportion of inter-individual variability remained unexplained. This indicates that additional factors beyond those measured in this study contribute to the physiology of posterior vitreous detachment. The use of cross-sectional data also precludes establishing temporal or causal relationships, and longitudinal analyses will be required to confirm the directionality of the associations observed. Additionally, the BCVA gradient observed in unadjusted comparisons likely reflects confounding by age and lens status, consistent with the lack of an independent association in the multivariable GEE model. Furthermore, residual confounding from ocular variables not captured in this study—such as refractive error, early phakic lens opacities, and subtle vitreoretinal interface abnormalities below the detection threshold of ultrasound and OCT—may also have influenced the observed associations. Although spherical equivalent was recorded, refractive error was not included as a covariate because axial length functioned as a more robust structural surrogate. Likewise, phakic lens opacity was not quantified beyond lens status, and therefore its potential contribution to BCVA variability or vitreous visibility cannot be fully excluded.

Second, the study population was derived from adults attending routine ophthalmologic evaluation at a tertiary center, which may introduce selection bias. Individuals seeking ophthalmic care tend to have higher comorbidity burden and may not fully represent the general population. While we excluded eyes with macular disease to obtain a physiologically “healthy” reference cohort, this approach may limit generalizability to community-based samples. Therefore, the findings should be interpreted as applicable to adults undergoing routine ophthalmic care in tertiary settings rather than to the general population. Accordingly, the values presented here should be interpreted as contextual reference information rather than definitive normative standards.

Third, genetic and molecular factors were not assessed. Variants in genes such as COL2A1 and COL4A1 [27], which participate in collagen structure and fibrillar organization, have been implicated in altered vitreous cohesion. Similarly, matrix-remodeling enzymes, including metalloproteinases, may contribute to vitreous liquefaction and posterior hyaloid instability [28,29].

In addition, individual anatomical characteristics—including vitreoretinal adhesion strength, scleral rigidity, and variation in intraocular pressure—may modulate the mechanical forces involved in PVD development [30]. Systemic conditions not captured in this dataset, such as dyslipidemia, autoimmune disorders or peripheral vasculopathies, could also influence vitreous aging.

Finally, environmental and lifestyle factors, such as ultraviolet radiation exposure or the practice of high-impact physical activities, have been related to accelerated ocular aging and a higher risk of vitreous alterations [31,32].

## 5. Conclusions

In this cohort of healthy adults without macular pathology, more advanced stages of posterior vitreous detachment were independently associated with longer axial length, systemic hypertension, previous cataract surgery, and older age. These findings underscore the combined influence of ocular biometrics, systemic vascular health and age-related factors on the progression of physiological vitreous separation in otherwise normal eyes.

Establishing reference vitreous patterns in healthy individuals provides a robust foundation for understanding the normal behavior of the vitreous gel and offers an objective framework for interpreting alterations of the vitreoretinal interface in disease. Quantitative characterization of vitreous status under physiological conditions represents a necessary step toward developing more refined classification schemes and improving clinical stratification strategies that incorporate vitreoretinal parameters.

## Figures and Tables

**Table 1:** Descriptive analysis of the study population (n = 340): baseline characteristics, comorbidities, and PVD distribution.

Characteristics	(n = 340)
Age (years)	55.5 ± 18.2
AXL (mm)	23.66 ± 1.46
BCVA (Snellen)	0.85 ± 0.26
Foveal thickness (µm)	277 ± 32
Female—n. (%)	233 (68.5)
Right eye—n. (%)	173 (50.9)
HTN—n. (%)	103 (30.3)
Diabetes—n. (%)	39 (11.5)
Collagen diseases—n. (%)	16 (4.7)
ASA	
1	209 (61.5)
2	123 (36.2)
3	8 (2.4)
Previous cataract surgery—n. (%)	67 (19.7)
Vitreous status (%)	
No PVD	105 (30.9)
Partial PVD	148 (43.5)
Complete PVD	87 (25.6)

Abbreviations: ASA: American Society of Anesthesiologists; AXL: axial length; BCVA: best-corrected visual acuity; HTN: arterial hypertension; PVD: posterior vitreous detachment.

**Table 2:** Demographic and clinical comparison in patients without macular disease according to PVD status (No PVD/Partial PVD/Complete PVD).

Characteristics	No PVD (n = 105)	Partial PVD (n = 148)	Complete PVD (n = 87)	*p*-Value
Age (years)	46.95 ± 14.96	53.93 ± 18.65	68.54 ± 13.30	<0.001
AXL (mm)	23.37 ± 1.30	23.68 ± 1.24	24.06 ± 1.90	0.022
BCVA (Snellen)	0.92 ± 0.15	0.85 ± 0.27	0.74 ± 0.32	<0.001
Foveal thickness (µm)	277 ± 24	277 ± 32	278 ± 40	0.966
Female—n. (%)	85 (81.0)	92 (62.2)	56 (64.4)	0.004
Right eye—n. (%)	55 (52.4)	80 (54.1)	38 (43.7)	0.287
HTN—n. (%)	8 (7.6)	36 (24.3)	59 (67.8)	<0.001
Diabetes—n. (%)	10 (9.5)	10 (6.8)	19 (21.8)	0.002
Collagen diseases—n. (%)	4 (3.8)	8 (5.4)	4 (4.6)	0.839
ASA				
1	83 (79.0)	100 (67.6)	26 (29.9)	
2	20 (19.0)	48 (32.4)	55 (63.2)	<0.001
3	2 (1.9)	0 (0)	6 (6.9)	
Previous cataract surgery—n. (%)	2 (1.9)	29 (19.6)	36 (41.4)	<0.001

Abbreviations: ASA: American Society of Anesthesiologists; AXL: axial length; BCVA: best-corrected visual acuity; HTN: arterial hypertension; PVD: posterior vitreous detachment.

**Table 3:** Clustered generalized estimating equations (GEE) ordinal logit model evaluating independent factors associated with increasing PVD severity. Exchangeable working correlation structure; robust (sandwich) standard errors. OR > 1 indicates higher odds of being in a more advanced PVD category.

Factor	OR	95% CI	*p*-Value
Age (per year)	1.03	1.01–1.05	0.012
AXL (per mm)	1.31	1.1–1.53	0.001
HTN	7.04	2.60–19.00	<0.001
Sex (female vs. male)	1.17	0.71–1.91	0.544
Diabetes mellitus	1.12	0.45–2.83	0.807
Prior cataract surgery	2.54	1.34–4.74	0.004
ASA score	1.26	0.59–2.79	0.569
BCVA	1.26	0.50–3.17	0.629

Abbreviations: ASA: American Society of Anesthesiologists; AXL: axial length; BCVA: best-corrected visual acuity; HTN: arterial hypertension.

## Data Availability

The data presented in this study are available on request from the corresponding author.

## Abbreviations

The following abbreviations are used in this manuscript:

AMD	Age-related Macular Degeneration
AREDS	Age-Related Eye Disease Study
ASA	American Society of Anesthesiologists
AXL	Axial Length
BCVA	Best-Corrected Visual Acuity
GEE	Generalized Estimating Equations
HA	Hyaluronic Acid
HTN	Hypertension
OCT	Optical Coherence Tomography
PVD	Posterior Vitreous Detachment

## References

[1] Sebag J. (1987). Structure, function, and age-related changes of the human vitreous. Vitr. Vitreoretin. Interface.

[2] Uchino E., Uemura A., Ohba N. (2001). Initial stages of posterior vitreous detachment in healthy eyes of older persons evaluated by optical coherence tomography. Arch. Ophthalmol..

[3] Kishi S., Demaria C., Shimizu K. (1986). Vitreous cortex remnants at the fovea after spontaneous vitreous detachment. Int. Ophthalmol..

[4] Riley R.D., Ensor J., Snell K.I.E., Harrell F.E., Martin G.P., Reitsma J.B., Van Smeden M. (2020). Calculating the sample size required for developing a clinical prediction model. BMJ.

[5] Johnson M.W. (2005). Perifoveal vitreous detachment and its macular complications. Trans. Am. Ophthalmol. Soc..

[6] Wang M.D., Truong C., Mammo Z., Hussnain S.A., Chen R.W.S. (2021). Swept Source Optical Coherence Tomography Compared to Ultrasound and Biomicroscopy for Diagnosis of Posterior Vitreous Detachment. Clin. Ophthalmol..

[7] Johnson M.W. (2010). Posterior vitreous detachment: Evolution and complications of its early stages. Am. J. Ophthalmol..

[8] Stefánsson E., Sebag J. (2014). Vitreous, oxygen, and the regulation of retinal metabolism. Vitreous: In Health and Disease.

[9] Favre M., Goldmann H. (1956). Genesis of posterior vitreus body detachment. Ophthalmologica.

[10] Foos R.Y. (1972). Vitreoretinal juncture; topographical variations. Investig. Ophthalmol..

[11] Hikichi T., Trempe C.L. (1994). Relationship between floaters, light flashes, or both, and complications of posterior vitreous detachment. Am. J. Ophthalmol..

[12] Ripandelli G., Coppe A.M., Parisi V., Olzi D., Scassa C., Chiaravalloti A., Stirpe M. (2007). Posterior vitreous detachment and retinal detachment after cataract surgery. Ophthalmology.

[13] Sebag J. (2008). Vitreoschisis. Graefe’s Arch. Clin. Exp. Ophthalmol..

[14] Yonemoto J., Ideta H., Sasaki K., Tanaka S., Hirose A., Oka C. (1994). The age of onset of posterior vitreous detachment. Graefe’s Arch. Clin. Exp. Ophthalmol..

[15] Machemer R., Buettner H., Norton E.W., Parel J.M. (1971). Vitrectomy: A pars plana approach. Trans. Am. Acad. Ophthalmol. Otolaryngol..

[16] Sebag J. (1998). Pharmacologic vitreolysis. Retina.

[17] Age-Related Eye Disease Study Research Group (2001). A randomized, placebo-controlled, clinical trial of high-dose supplementation with vitamins C and E and beta carotene for age-related cataract and vision loss: AREDS report no. 9. Arch. Ophthalmol..

[18] Ito Y., Terasaki H., Suzuki T., Kojima T., Mori M., Ishikawa K., Miyake Y. (2003). Mapping posterior vitreous detachment by optical coherence tomography in eyes with idiopathic macular hole. Am. J. Ophthalmol..

[19] Gupta P., Sadun A.A., Sebag J., Sebag J. (2008). Vitreomacular traction syndrome. Vitreous: In Health and Disease.

[20] Henderson B.A., Kim J.Y., Ament C.S., Ferrufino-Ponce Z.K., Grabowska A., Cremers S.L. (2007). Clinical pseudophakic cystoid macular edema. Risk factors for development and duration after treatment. J. Cataract. Refract. Surg..

[21] Ong Y.T., Wong T.Y., Klein R., Klein B.E., Mitchell P., Sharrett A.R., Ikram M.K. (2013). Hypertensive retinopathy and risk of stroke. Hypertension.

[22] Wong T.Y., Klein R., Klein B.E., Tielsch J.M., Hubbard L., Nieto F.J. (2001). Retinal microvascular abnormalities and their relationship with hypertension, cardiovascular disease, and mortality. Surv. Ophthalmol..

[23] Wong T.Y., Mitchell P. (2007). The eye in hypertension. Lancet.

[24] Tso M.O., Jampol L.M. (1982). Pathophysiology of hypertensive retinopathy. Ophthalmology.

[25] Hilford D., Hilford M., Mathew A., Polkinghorne P.J. (2009). Posterior vitreous detachment following cataract surgery. Eye.

[26] Ivastinovic D., Schwab C., Borkenstein A., Lackner E.M., Wedrich A., Velikay-Parel M. (2012). Evolution of early changes at the vitreoretinal interface after cataract surgery determined by optical coherence tomography and ultrasonography. Am. J. Ophthalmol..

[27] Richards A.J., Martin S., Yates J.R., Scott J.D., Baguley D.M., Pope F.M., Snead M.P. (2000). COL2A1 exon 2 mutations: Relevance to the Stickler and Wagner syndromes. Br. J. Ophthalmol..

[28] Jin M., Kashiwagi K., Iizuka Y., Tanaka Y., Imai M., Tsukahara S. (2001). Matrix metalloproteinases in human diabetic and nondiabetic vitreous. Retina.

[29] Brown D.J., Bishop P., Hamdi H., Kenney M.C. (1996). Cleavage of structural components of mammalian vitreous by endogenous matrix metalloproteinase-2. Curr. Eye Res..

[30] Kishi S., Shimizu K. (1990). Posterior precortical vitreous pocket. Arch. Ophthalmol..

[31] Balazs E.A. (1954). Studies on the structure of the vitreous body. I. The absorption of ultraviolet light. Am. J. Ophthalmol..

[32] Marshall J. (1985). Radiation and the ageing eye. Ophthalmic Physiol. Opt..

